# Phylogenetic test of speciation by host shift in leaf cone moths (*Caloptilia*) feeding on maples (*Acer*)

**DOI:** 10.1002/ece3.2266

**Published:** 2016-06-21

**Authors:** Ryosuke Nakadai, Atsushi Kawakita

**Affiliations:** ^1^ Center for Ecological Research Kyoto University Hirano 2‐509‐3 Otsu Shiga 520‐2113 Japan

**Keywords:** Diversification, herbivorous insect, host plant, host shift, speciation

## Abstract

The traditional explanation for the exceptional diversity of herbivorous insects emphasizes host shift as the major driver of speciation. However, phylogenetic studies have often demonstrated widespread host plant conservatism by insect herbivores, calling into question the prevalence of speciation by host shift to distantly related plants. A limitation of previous phylogenetic studies is that host plants were defined at the family or genus level; thus, it was unclear whether host shifts predominate at a finer taxonomic scale. The lack of a statistical approach to test the hypothesis of host‐shift‐driven speciation also hindered studies at the species level. Here, we analyze the radiation of leaf cone moths (*Caloptilia*) associated with maples (*Acer*) using a newly developed, phylogeny‐based method that tests the role of host shift in speciation. This method has the advantage of not requiring complete taxon sampling from an entire radiation. Based on 254 host plant records for 14 *Caloptilia* species collected at 73 sites in Japan, we show that major dietary changes are more concentrated toward the root of the phylogeny, with host shift playing a minor role in recent speciation. We suggest that there may be other roles for host shift in promoting herbivorous insect diversification rather than facilitating speciation per se.

## Introduction

Herbivorous insects comprise one of the major components of earth's biodiversity. Because the diversity of herbivorous insects is often correlated with host plant diversity (Lawton and Schroeder 1977; Wiegmann et al. 2002; Janz et al. 2006; Joy and Crespi 2012; Ferrer‐Paris and Sánchez‐Mercado 2013; Isaka and Sato 2015; Lin et al. 2015), the cycle of host plant adaptation and host plant shift is commonly invoked as the major process generating high diversity (Mitter and Brooks 1983; Craig et al. 2001; Wheat et al. 2007; Futuyma and Agrawal 2009; Bennett and O'Grady 2012). For example, a classical study by Farrell (1998) showed that herbivorous insects using angiosperms as hosts are more species rich than those using gymnosperms among the Phytophaga beetles, suggesting that the diversity of angiosperms has facilitated speciation by host shift in the beetles that feed on them. Studies of host races in herbivorous insects showed that specialization to a novel host plant sometimes results in reproductive isolation between insects using different hosts (Feder et al. 1988; Groman and Pellmyr 2000; Hawthorne and Via 2001; Nosil et al. 2002; Thomas et al. 2003; Malausa et al. 2005; Ohshima 2012; Xue et al. 2014), providing a mechanistic explanation of how host shifts may promote speciation. Understanding the role of host plant shifts in generating diversity is thus a current focus in the study of herbivorous insect diversification (Marvaldi et al. 2002; Stireman et al. 2005; Wheat et al. 2007; Winkler et al. 2009; Fordyce 2010; Funk 2010; Matsubayashi et al. 2010; Nyman 2010; Soria‐Carrasco et al. 2014).

However, phylogenetic analyses of herbivorous insect radiation have often demonstrated conservatism in host plant use by herbivorous insects (Crespi et al. 1998; Lopez‐Vaamonde et al. 2003; Wahlberg 2007; Winkler and Mitter 2008; Nyman et al. 2010; Jousselin et al. 2013; Doorenweerd et al. 2015). For example, Nyman et al. (2010) showed that only 20% of the speciation events in nematine sawflies were accompanied by shifts between host plant families, and Doorenweerd et al. (2015) showed that host use was generally conserved at the plant family level, with biogeographic processes playing a greater role in the recent speciation of nepticulid moths. Extreme cases of host plant conservatism are found in gall wasps feeding on oaks (Stone et al. 2009) or micropterigid moths that have radiated on a single liverwort species (Imada et al. 2011). However, many phylogenetic studies that tested for host conservatism defined host plants at the plant family or genus level (Lopez‐Vaamonde et al. 2003; Wahlberg 2007; Nyman et al. 2010; Jousselin et al. 2013; Doorenweerd et al. 2015). The relative importance of host shifts in herbivorous insect speciation should ideally be assessed using species‐level phylogenies with data on all known host associations.

Two major obstacles hamper analysis at the species level. First, because most radiations of herbivorous insect groups occur at the continental scale, it is usually difficult to achieve complete taxon sampling while having host association data for each species. It is therefore not surprising that some of the best‐sampled phylogenies are those for less mobile herbivorous insect groups (e.g., Imada et al. 2011). Second, an appropriate method of analyzing host plant shifts along phylogenies has been lacking. Coding host plant associations at the family or genus level would simplify analysis because methods such as ancestral character state reconstructions are then applicable. However, many herbivorous insects use several closely related plant species (i.e., polyphagy) with varying levels of preference (Smiley 1978; Roininen and Tahvanainen 1989; Thompson 1998; Scheirs et al. 2000; D'Costa et al. 2013; Nakadai and Murakami 2015), which complicates analysis of the ancestral state regarding host use. In addition, individual host plant species cannot be considered as discrete character states because they are phylogenetically nonindependent (Pearse and Altermatt 2013). Ideally, the dissimilarity of host use between a pair of herbivorous insect species should be weighed by the phylogenetic disparity of the host plants.

In this study, we assess the importance of host shifts in the speciation process of herbivorous insects by developing a new method that overcomes these issues. This method focuses on whether host plant shifts are concentrated toward the roots or the tips of the insect phylogenetic tree, while taking into account host plant phylogeny in the calculation of host use dissimilarity between a pair of herbivorous insect species. If most speciation events are associated with host shifts, the level of disparity in host use between a pair of herbivorous insect species will on average be greater for phylogenetically more closely related pairs (Fig. 2A). Alternatively, if most host shifting events occurred during the initial stage of the radiation and more recent speciation events were independent of host shifts, the level of difference in host use would be larger toward the root of the phylogenetic tree (Fig. 2B). We focused on the interaction between a group of leaf cone moths (*Caloptilia*, Gracillariidae) and their maple hosts (*Acer*, Sapindaceae). The *Caloptilia*–*Acer* interaction is appropriate for testing host‐shift‐driven speciation at fine taxonomic scales because a previous study demonstrated large variation in the pattern of host use among *Caloptilia* species (Nakadai and Murakami 2015). The genus *Acer* is one of the most taxonomically diverse groups of trees in the Northern Hemisphere, particularly in the temperate regions of East Asia, eastern North America, and Europe (van Gelderen et al. 1994). The genus comprises 124 species in the Northern Hemisphere, 81% of which are distributed in China, Korea, and Japan (Renner et al. 2007). A previous taxonomic study of *Caloptilia* identified 11 species associated with *Acer* in Japan alone, which have high morphological affinity to each other (Kumata 1982). Based on extensive geographic sampling, we establish full host plant records for these 11 species and three newly found ones, and analyze them using the above method to assess the relative importance of host shift in the speciation of *Caloptilia* moths feeding on *Acer* trees.

## Materials and Methods

### Study material

The genus *Caloptilia* is globally distributed and includes nearly 300 described species, of which 27 feed on maples (De Prins and De Prins 2015). In Japan, 51 species are described feeding on 21 host plant families, and 11 of them use *Acer*, which is the most common host plant genus of Japanese *Caloptilia* (Kumata et al. 2013). The feeding habits of the larvae change dramatically between the early and late developmental stages. Upon hatching, larvae mine the surface layer of the leaf (i.e., leaf miners) until the third instar, then exit the mine, and form the edge of the leaf into a roll within which they feed externally until the final instar (hence the name leaf cone moth) (Kumata et al. 2013). Some species are leaf gallers or blotch miners at the final instar and do not roll leaves. Each species is usually associated with a single plant genus.

### Sampling, DNA sequencing, and phylogenetic analyses

**Figure 1:**
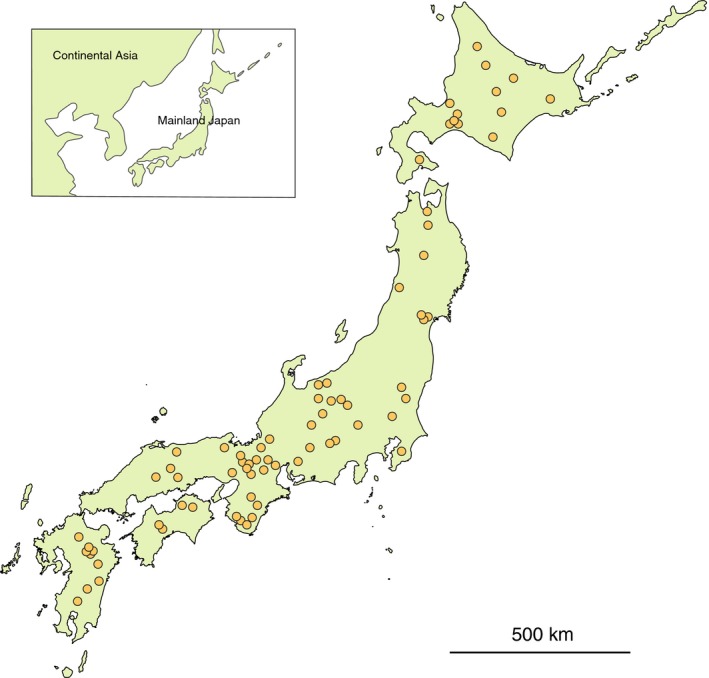
Sampling localities of *Caloptilia* moths collected from *Acer* trees in Japan. Sampling information for each species shown in Figure S2.

We sampled *Caloptilia* moths that use *Acer* trees at 73 sites covering a wide geographic range in Japan (Figs. 1, S2) during May–October of 2011–2015. Moths were sampled by searching for larvae in leaf rolls (fourth or fifth instar) or pupae on maple leaves. In total, 254 specimens were obtained, used to delimit species and to establish the host range for each species. Delimitation of species was based on sequences of the mitochondrial cytochrome oxidase subunit I (COI) gene; major divergences in COI sequences clearly corresponded with differences in wing pattern and genital morphology. Species were morphologically identified following Kumata (1982). To further determine whether the *Caloptilia* species feeding on maples resulted from a single radiation, we additionally sampled 44 *Caloptilia* species that use nonmaple hosts and six species in closely related genera (*Gracillaria*,* Calybites*, and *Eucalybites*; for details, see Table S1) and reconstructed a species‐level phylogeny of *Caloptilia*. For the species‐level phylogeny, one representative specimen of each *Caloptilia* species feeding on maple was included in the analysis. All moth specimens were kept in ethanol prior to DNA extraction.

We extracted genomic DNA using the NucleoSpin Tissue Kit (Macherey‐Nagel, Düren, Germany). The head capsule of the larva or the head, wings, and abdomen of the adult were stored as vouchers. The COI gene was sequenced for all of the 254 moths collected from maples. For the species‐level phylogenetic analysis, we sequenced four genomic regions: COI and the nuclear arginine kinase (ArgK), carbamoyl‐phosphate synthetase 2 (CAD), and elongation factor 1‐alpha (EF‐1*α*) genes. We designed new primer sets for ArgK, CAD, and EF‐1*α* (Table S3) based on sequences available for other species of Gracillariidae in the database. The information on existing primer sets for CO1 and EF‐1*α* is also provided in Table S3. Polymerase chain reaction (PCR) amplifications were carried out under the following conditions: initial denaturation step at 94°C for 5 min; 30 cycles of 94°C for 30 sec, 50°C for 30 sec, and 72°C for 1 min; and a final extension at 72°C for 7 min. Products were sequenced on an ABI 3100 automated sequencer using BigDye chain termination chemistry (Applied Biosystems, Foster City, CA), and obvious sequence errors were manually corrected using MEGA 6.06 (Tamura et al. 2013). Obtained sequences were aligned using Mafft ver. 6.901 (Katoh and Toh 2008) under the default settings. The resulting dataset contained 658, 573, 614, and 541 base pairs of COI, ArgK, CAD, and EF‐1*α*, respectively. Species‐level phylogenetic trees were constructed using two datasets: (1) an all‐genes dataset (COI + ArgK + CAD + EF‐1*α*) and (2) a nuclear‐only dataset (ArgK + CAD + EF‐1*α*). The latter was created because a previous phylogenetic study of Gracillariidae suggested that nuclear genes provide strong phylogenetic signals at the genus and species levels (Kawahara et al. 2011). We reconstructed phylogenetic trees by maximum‐likelihood and Bayesian methods for each dataset. The maximum‐likelihood analysis was performed using RAxML ver. 8.0 (Stamatakis 2014). We conducted 100 replicates of shotgun search for the likelihood ratchet and assessed nodal support using bootstrap analyses with 1000 replications. We also conducted Bayesian phylogenetic analysis using MrBayes5D (Tanabe 2008), a modified version of MrBayes3.1.2 (Ronquist and Huelsenbeck 2003). We used the following settings for the Bayesian analysis: number of Markov chain Monte Carlo generations, five million; sampling frequency, 100; and burn‐in, 5001. The burn‐in size was determined by checking the convergence of log likelihood (ln L) plotted against generation time. In both methods, we used Kakusan4 (Tanabe 2011) to determine appropriate models of sequence evolution under the BIC4 criterion.

### Hypothesis and randomization tests for validation

**Figure 2:**
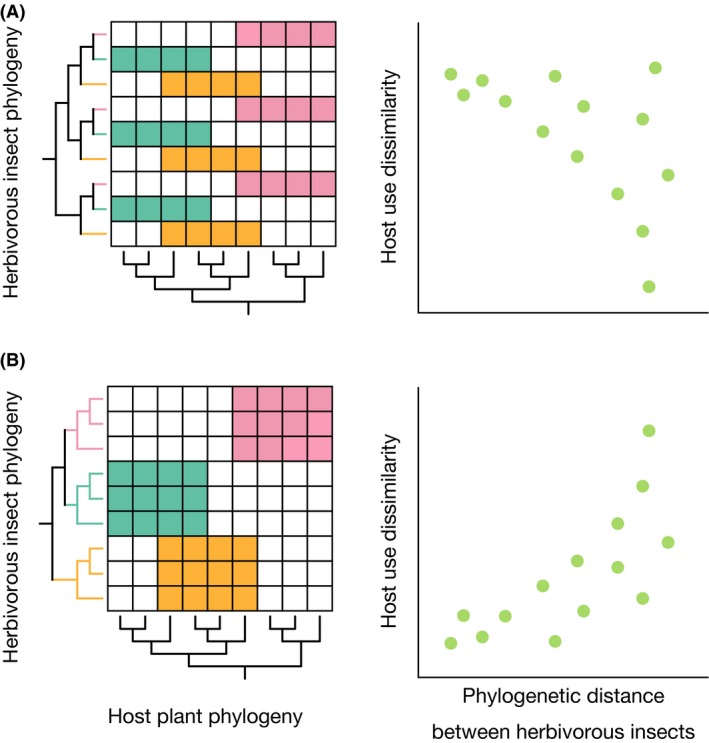
Phylogenetic distributions of host use arising from different speciation modes in herbivorous insects. (A) Distribution of host use on the phylogeny of a hypothetical insect group in which speciation is mainly associated with host shifts. (B) Distribution of host taxa when speciation mainly involves other processes without host shifts.

To test the relative importance of host shift in the speciation process from phylogeny, we assumed two contrasting scenarios (Fig. 2). If most speciation events are associated with host shifts, the dissimilarity in host use will on average be larger for phylogenetically more closely related pairs of *Caloptilia* moths (Fig. 2A). Conversely, if most speciation events occur during the initial stage of the radiation and more recent speciation events are independent of host shifts, host use dissimilarity will be larger for phylogenetically more distantly related pairs of *Caloptilia* moths (Fig. 2B). A similar framework was proposed by Nyman et al. (2010), but their method cannot be applied to species‐level analysis. Following Barraclough et al. (1999), we used randomizations to compare the observed pattern of host use to that expected under a null model of no association with cladogenesis. Our null model hypothesized that changes occurred at random and independently across the tree. The statistic used to test the association between phylogenetic distance and the degree of difference in host use is expressed as the sum across all nodes of phylogenetic distance *X*_*i*_ multiplied by the degree of host use dissimilarity *H*_*i*_ (see the next section for detailed calculation of dissimilarity),

$$\sum_{i=1}^{i=m}X_{i}H_{i}.$$

If differences in host use are greater between closely related species, the above statistic is expected to be smaller than that under the null model and vice versa. Thus, we tested for a significant concentration of changes toward either the tips or the root of the tree. A positive sign indicates the concentration of changes toward the tips, whereas a negative sign indicates that more changes occurred toward the root. The null distribution was obtained by randomly shuffling observed changes among branches of the tree and calculating the above statistic in each null trial. The two‐tailed probability of the observed value was calculated based on 10,000 randomizations. A similar randomization method was used by Barraclough et al. (1999) and Sauer and Hausdorf (2009) to study adaptive character evolution in tiger beetles and land snails, respectively.

In addition, we calculated the standardized effect size (SES) as the observed test statistic minus the mean of the null distribution, divided by the standard deviation of the null distribution. This null model approach is commonly used for expressing biological differences regardless of the units of the indices (McCabe et al. 2012).

### Indices of dissimilarity in host use

**Figure 3:**
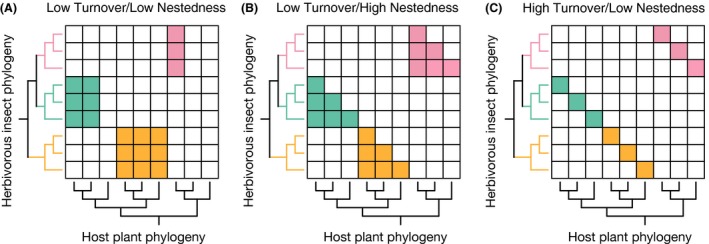
Possible patterns of plant–herbivore association. (A) Low turnover/low nestedness, (B) low turnover/high nestedness, and (C) high turnover/low nestedness. Both Jaccard and Unifrac indices perform similarly in (A) and (B), whereas in (C), the nestedness component of the Unifrac index between a pair of closely related herbivores will be lower than that of the Jaccard index. This is because host use is similar when host phylogeny is taken into account but maximally dissimilar in the absence of host phylogenetic information.

We used both Jaccard (Jaccard 1912; Koleff et al. 2003) and Unifrac (Lozupone and Knight 2005) indices to quantify the degree of difference in host use between a pair of *Caloptilia* moths feeding on *Acer* trees. Both indices are commonly used in community ecology for assessing the degree of dissimilarity between two communities (Cavender‐Bares et al. 2009). The Unifrac index is analogous to the Jaccard dissimilarity index, but takes into account phylogenetic information (Lozupone and Knight 2005), which in the present case is the plant phylogeny. The Unifrac index has an advantage over the Jaccard index especially when there is missing information on host association; the latter index assumes an equal weight for all host plant species, whereas the former weighs host plants according to their phylogenetic relatedness and is thus less sensitive to missing data. In this study, we used the phylogeny of 30 Japanese *Acer* species published by Nakadai et al. (2014). In addition, both Jaccard and Unifrac indices can be partitioned into two components of dissimilarity: turnover and nestedness (Baselga 2010; Leprieur et al. 2012). In community ecology, the turnover of a species assemblage refers to the replacement of some species by others as a consequence of historical events, such as geographic barrier formation or environmental sorting (Baselga 2010). In contrast, the nestedness of a species assemblage occurs when the species composition of sites with a smaller number of the species is a subset of that of species‐rich sites, which reflects a spatial pattern of species loss resulting from dispersal limitation or environmental filtering (Hirao et al. 2015). In our study, the turnover component indicates the degree of nonoverlapping host use, and the nestedness component represents the difference in the degree of specialization between insect species with shared host plants (Fig. 3). All indices were calculated using the “betapart” package (Baselga and Orme 2012) in R ver. 3.2.2 (R Core Team 2015).

## Results

**Figure 4:**
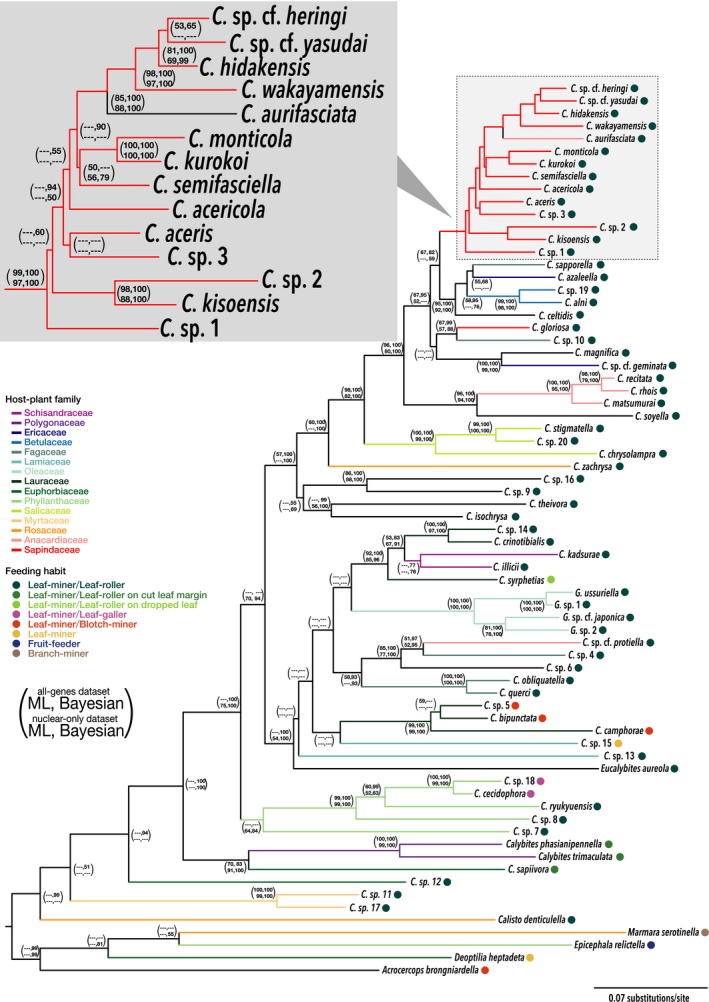
Phylogeny of *Caloptilia* moths and their related groups. The phylogeny was constructed by maximum‐likelihood method using four genomic regions (COI, ArgK, CAD, and EF‐1*α*) of 71 species.

**Figure 5:**
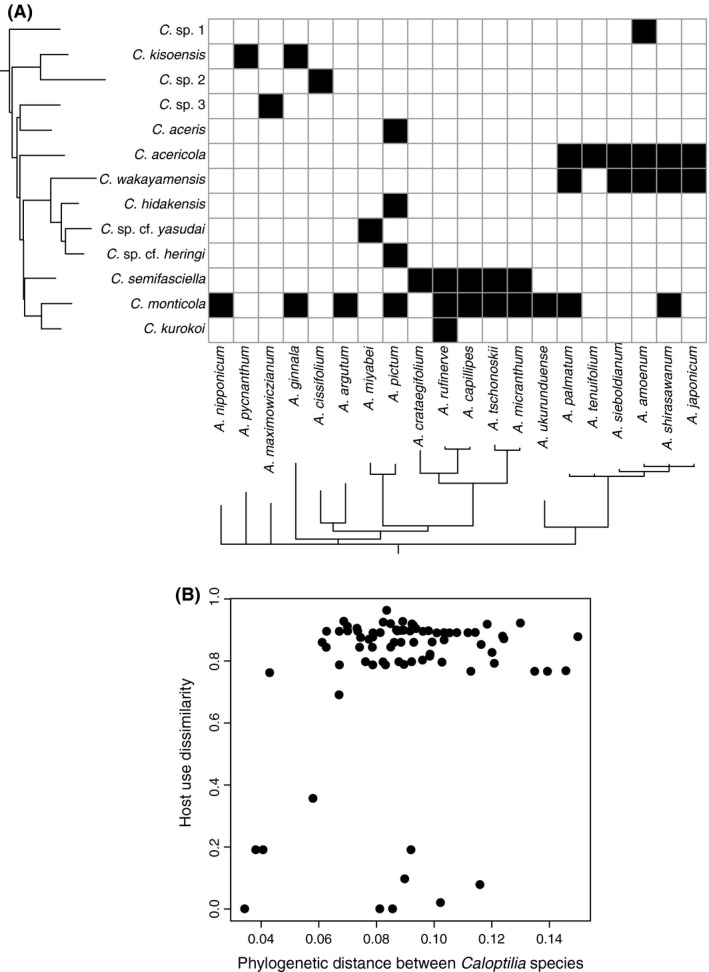
The results of *Acer*–*Caloptilia* interactions obtained from wide range sampling in Japan. (A) Phylogram of 13 species of *Caloptilia* pruned from a phylogeny of this genus and related groups (Table S3) and a phylogram of 20 species of *Acer* trees pruned from a phylogeny of this genus in Japan (Nakadai et al. 2014). The complete phylogeny of *Acer* trees was the 50% majority‐rule consensus of trees sampled from the stationary distribution of a Bayesian analysis of four chloroplast DNA loci sampled from 30 species, including some varieties. (B) The plot of phylogenetic distance between *Caloptilia* moths (all‐genes dataset) versus host use dissimilarity (turnover and nestedness components of the Unifrac index).

Extensive sampling of *Caloptilia* moths throughout Japan identified 14 species feeding on maples (Figs. 4, S1), of which three were newly discovered in this study. This represents ca. 40% of the *Caloptilia* species known to feed on maples (De Prins and De Prins 2015). Most species were widely distributed throughout the range, although some were only found at a limited number of sites (Fig. S2). Some species were apparently specialists on single *Acer* species (e.g., *Caloptilia hidakensis*,* Caloptilia kurokoi*), whereas others were collected from many hosts. Overall, each species uses 1–11 *Acer* species, with a mean of 3.0 ± 3.0 (Fig. 5).

Species‐level phylogenetic analyses based on 2386 bp of the combined COI, ArgK, CAD, and EF‐1*α* dataset produced a well‐resolved phylogeny (Fig. 4). All of the *Caloptilia* species feeding on *Acer* were closely related, although they were not monophyletic. One species, *Caloptilia gloriosa*, was positioned outside of the clade consisting mainly of *Acer*‐feeding *Caloptilia* (Fig. 4), and another species, *Caloptilia aurifasciata*, feeding on *Toxicodendron* (Anacardiaceae), was embedded within this clade (Fig. 4). We thus focused on the clade containing *C. aurifasciata* and the 13 species feeding on *Acer* for the analysis of host shifts. We conducted randomization tests separately for datasets with and without *C. aurifasciata*. Because information on the phylogenetic distance between *Acer* and *Toxicodendron* (the host of *C. aurifasciata*) was not available, we assumed the maximum turnover (1) and minimum nestedness (0) for the calculation of dissimilarity indices between *C. aurifasciata* and *Acer*‐feeding *Caloptilia*.

The results of randomization tests indicated that the turnover components and the combined turnover and nestedness components of both Jaccard and Unifrac indices are greater between distantly related species than expected under the null model (positive signs in Table 1), although the trend was not significant for the Jaccard index except for the turnover component of the all‐genes dataset. The nestedness component showed negative signs but was not statistically significant (Table 1). These results support the hypothesis of phylogenetic conservatism in host use (Fig. 2B). Inclusion of *C. aurifasciata*, which feeds on *Toxicodendron*, did not change the overall pattern but slightly strengthened the trend, with tests using both Jaccard and Unifrac indices becoming significant (Table S4).

**Table 1:** Relationships between differences of host use and phylogenetic distance between *Caloptilia* species feeding on *Acer* according to randomization tests

Dataset		Turnover + nestedness			Turnover			Nestedness		
		Sign	SES		Sign	SES		Sign	SES	
All‐genes dataset	Jaccard index	+	1.66	n.s.	+	1.95	*	–	−1.26	n.s.
	Unifrac index	+	2.17	*	+	2.16	*	–	−0.85	n.s.
Nuclear‐only dataset	Jaccard index	+	1.90	n.s.	+	1.95	n.s.	–	−1.10	n.s.
	Unifrac index	+	2.72	**	+	2.40	*	–	−0.60	n.s.

Positive signs of differences in host use with phylogenetic distance suggest that changes are concentrated toward the root and negative signs suggest that changes occur near the tips. Significance level: n.s., *P *≥* *0.05; *P <* *0.05; **P <* *0.01.

The SES values provide a quantitative measure of the strength of association between host use dissimilarity and phylogenetic distance (Table 1). Overall, the values for the turnover component and the combined turnover + nestedness component were greater when host plant phylogeny was taken into account (Unifrac index) than when it was not (Jaccard index).

## Discussion

### Application of randomization test in the study of herbivorous insect speciation

In this article, we describe a new method for testing the role of host shift in herbivorous insect speciation. We identified three beneficial features of this method. First, it is less sensitive to incomplete species sampling. It is usually difficult to sample every species for the entire radiation (Lopez‐Vaamonde et al. 2003; Nyman et al. 2006; Agrawal and Fishbein 2008; Stone et al. 2009; Doorenweerd et al. 2015), and conventional methods of analyzing the effects of host shifts on phylogeny (e.g., ancestral character state reconstruction) are sensitive to species sampling. However, because our analysis focuses on whether host use changes are concentrated toward either the root or the tips of the phylogenetic tree, complete sampling is not required as long as species sampling is not biased (e.g., toward species feeding only on a particular species of host).

Second, the method permits analysis of speciation by host shift at a broader geographic scale. In many cases, herbivorous insect species have broader distributions than individual host plant species, so sister herbivore species occurring in allopatry should always use different hosts, even if diet shift was not the major cause of speciation. The use of a dissimilarity index controlling for host phylogeny partly remedies this problem (Pearse and Altermatt 2013; Pearse et al. 2013) because related plant species are generally similar in their traits associated with susceptibility to herbivores (Rasmann and Agrawal 2011; D'Costa et al. 2014; Nakadai and Murakami 2015), and thus host use dissimilarity will consistently be low if no major diet shift has occurred during speciation. Caution is needed in cases where the group of herbivores being studied has extremely high or low host specificity because, in both cases, the method may overestimate host use conservatism.

Finally, calculation of SES allows comparison of trends among different studies (McCabe et al. 2012) because SES is independent of differences in the number of herbivore species included in the dataset. Previous phylogenetic studies assessed the percentage of host shifts between host plant families in each taxonomic group (Lopez‐Vaamonde et al. 2003; Nyman et al. 2010; Doorenweerd et al. 2015), but quantitative comparisons among studies were difficult due to the lack of a standardized measure for comparison.

We note that our method has a link to those developed previously to test the degree of cospeciation between a pair of host and parasite. However, because they are designed to test for cospeciation, they either assume that each parasite is associated with only one host at any given time (Page 1994; Ronquist 1995; Charleston and Robertson 2002; Merkle and Middendorf 2005; Conow et al. 2010) or that host and parasite speciation events are simultaneous in time (Legendre et al. 2002), which are not realistic for many plant–herbivore associations. Recently, Rafferty and Ives (2013) and Hadfield et al. (2014) developed methods that do not require such assumptions and uses GLMM to test for interaction effect of two phylogenies, but the methods are not designed to test the polarity of trait divergence occurring either toward the tips or the root of the phylogeny as in our method.

One weakness of our analysis is that we treated host association based on presence/absence, but in reality, preference levels are not equal for all of the host plant species observed. We could not quantify host preference in this study because it is necessary to standardize both sampling effort and host abundance to obtain a comparative measure of host preference, which was difficult to accomplish at all sampling sites. However, the above‐described method can easily incorporate host preference when such data are available, as dissimilarity measures (Unifrac and Jaccard indices) are also designed for quantitative data. The newly developed method is presently intended for testing host‐shift‐driven speciation in herbivorous insects, but the overall framework is applicable, in principle, to studies of other types of ecological speciation. The source code for running the analysis in R is provided as Data S4. The source code and datasets for running the analysis in R is provided as Data S1–4.

### Alternative hypothesis on the speciation process of leaf cone moths feeding on maples

Application of the present method to the 13 species of maple‐feeding leaf cone moths suggested that major dietary changes are concentrated toward the root of the herbivore phylogenetic tree (Table 1). Because the Unifrac index takes into account plant phylogeny whereas the Jaccard index does not, significant positive sign for the Unifrac index and lack of significance for the Jaccard index indicate that the trend exists only when host plant phylogeny is taken into account in the calculation of dissimilarity. Thus, the results indicate that major dietary shifts play a minor role in recent speciation events, but shifts between very closely related hosts may have took place during recent *Caloptilia* speciation. The addition of *C. aurifasciata* generally strengthened the trend for both Jaccard and Unifrac indices because *C. aurifasciata* diverged from all other species toward the root of the tree and has a completely different diet. The Jaccard test, which was only marginally insignificant in the absence of *C. aurifasciata*, became significant after the inclusion of this species (Table S4).

Although our test indicated that speciation assisted by host shift may be relatively minor in this group, we do not deny the importance of major dietary changes as such events occur in some of the earliest speciation events. Nevertheless, host‐shift‐driven speciation may not be as important as commonly thought in generating the current diversity of *Caloptilia*. Because our analysis only tests for patterns, the alternative process that drives speciation in *Caloptilia* cannot be inferred from our data. However, previous studies proposed several possible processes by which herbivorous insects speciate without changing their diet (Imada et al. 2011; Bennett and O'Grady 2012; Yamamoto and Sota 2012; Hamm and Fordyce 2015). For some phytophagous insect groups, allopatric speciation without host shift may be a major factor causing radiation (Nyman et al. 2010; Imada et al. 2011), but in the case of Japanese leaf cone moths, the pattern is unclear based on visual inspection of the current geographic distribution (Fig. S1). Ecological shift within a host plant is also a significant process (Condon and Steck 1997; Cook et al. 2002; Joy and Crespi 2007; Althoff 2014; Mishima et al. 2014). For example, Zhang et al. (2015) demonstrated divergence induced by host plant ages in sympatric sister beetles (*Pyrrhalta maculicollis* and *Pyrrhalta aenescens*) feeding on elm. There is clearly a need to sample from a broader geographic area and to collect additional information on microniche divergence among leaf cone moths to fully understand the process underlying their diversification. Adding timeline to the divergence events of both herbivores and host plants should also facilitate the understanding of the role of host shift in herbivore radiation.

### Revealing the role of host shifts in herbivorous insect diversification

Our study proposed a method for assessing the relative importance of host shifts in herbivorous insect speciation. This method allows quantitative analysis at a fine taxonomic scale, but because we only applied it to one herbivorous insect group, the application of this method to various herbivorous insect groups will facilitate a more general discussion on herbivorous insect diversification. If host‐shift‐driven speciation turns out to be relatively minor in recent speciation, there may be another role for host shifts in promoting herbivorous insect diversification rather than facilitating speciation per se, such as facilitating the entry into novel niche spaces (Janzen 1968) and the coexistence of already diverged species (Rabosky 2009). Information on the phylogenetic pattern of host use is clearly increasing rapidly, and a standardized method would link studies using different systems and facilitate our understanding of the effects of host shift on herbivorous insect diversity.

## Conflict of Interest

None declared.

## Data Archiving

Obtained DNA sequences have been deposited in the DDBJ database under accession numbers LC127539–LC128013. Nucleotide alignments will be archived in TreeBase.

## Supporting information


**Figure S1.** Phylogeny of *Caloptilia* moths feeding on *Acer* based on mitochondrial COI with information on sampling site.

**Figure S2.** Distributions of 14 *Caloptilia* moth species feeding on *Acer*.

**Table S1.** Specimen information.
**Table S2.** DDBJ accession numbers.
**Table S3.** Primers used in this study.
**Table S4.** The results of randomization tests including *C. aurifasciata* that feeds on *Toxicodendron*.

**Data S1.** Newick format data containing phylogenetic information of 13 *Caloptilia* moths feeding on *Acer* trees.

**Data S2.** Newick format data containing phylogenetic information of 20 *Acer* species.

**Data S3.** CSV format file containing host use information.

**Data S4.** Text file containing the command for running the randomization analysis in R using Data S1‐3.
