# Supplementary figures and images for: Phylogenetic test of speciation by host shift in leaf cone moths (Caloptilia) feeding on maples (Acer)
Source: Ecol Evol. 2016 Jun 21;6(14):4958–70. doi: 10.1002/ece3.2266 (PMC4979720; doi:10.1002/ece3.2266)

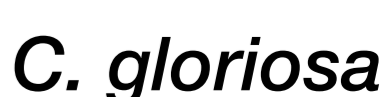

Supplement: Supplementary file 1 — Figure S1. Phylogeny of Caloptilia moths feeding on Acer based on mitochondrial COI with information on sampling site. [file ECE3-6-4958-s001.pdf]
