# Supplementary material for: Phylogenetic test of speciation by host shift in leaf cone moths (Caloptilia) feeding on maples (Acer)
Source: Ecol Evol. 2016 Jun 21;6(14):4958–70. doi: 10.1002/ece3.2266 (PMC4979720; doi:10.1002/ece3.2266)

(a) *C. acericola*

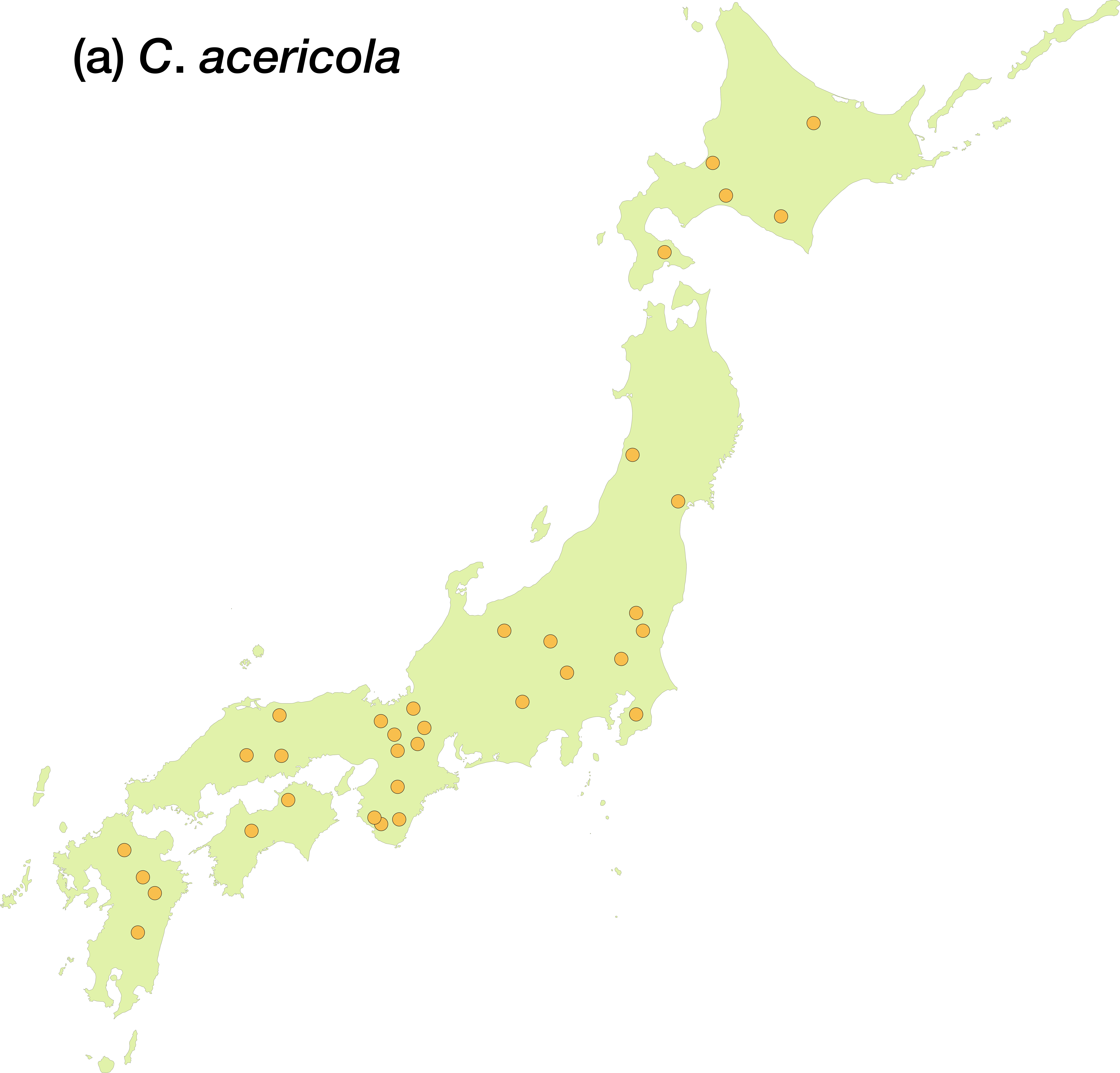

(b) *C. aceris*

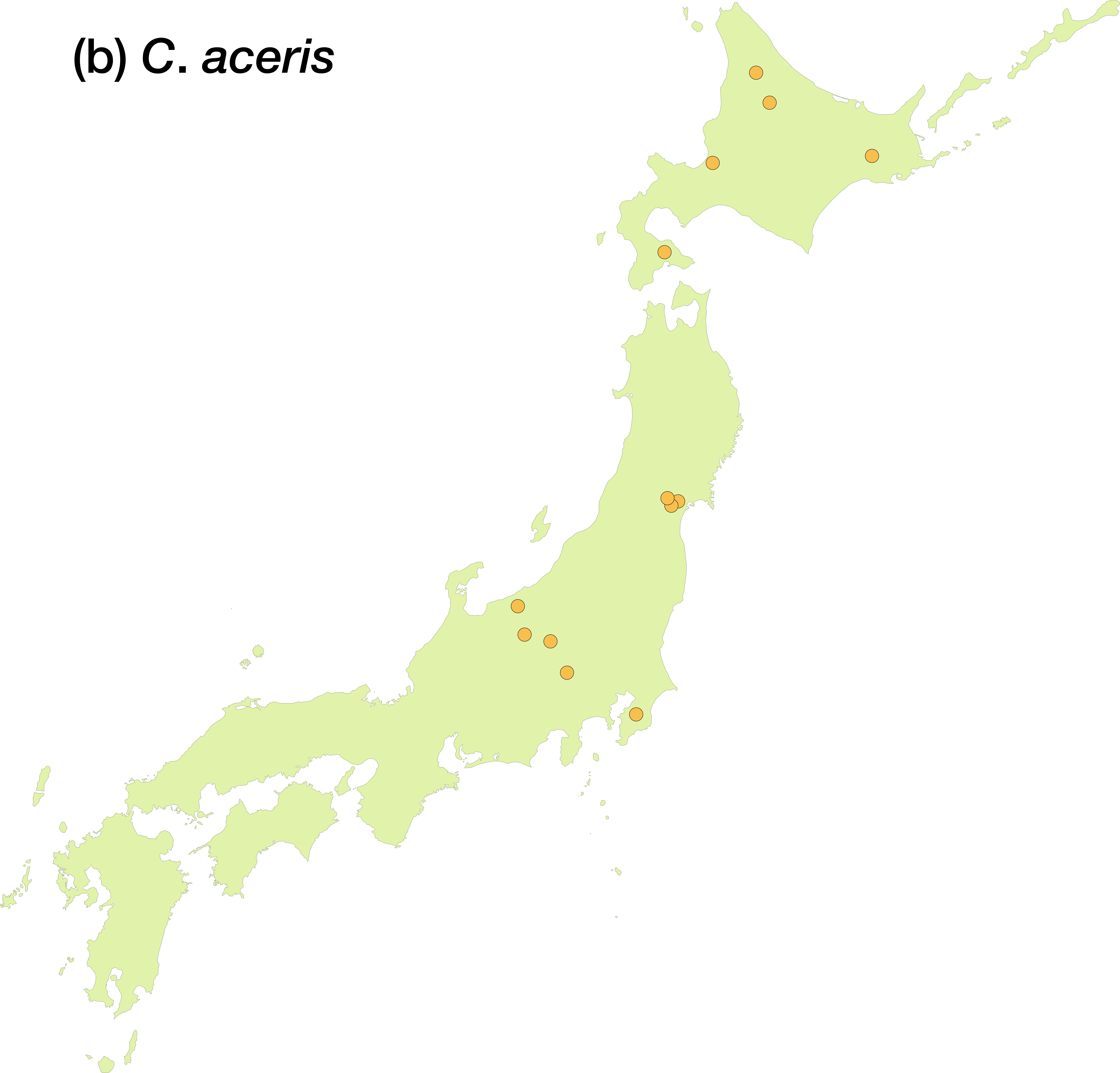

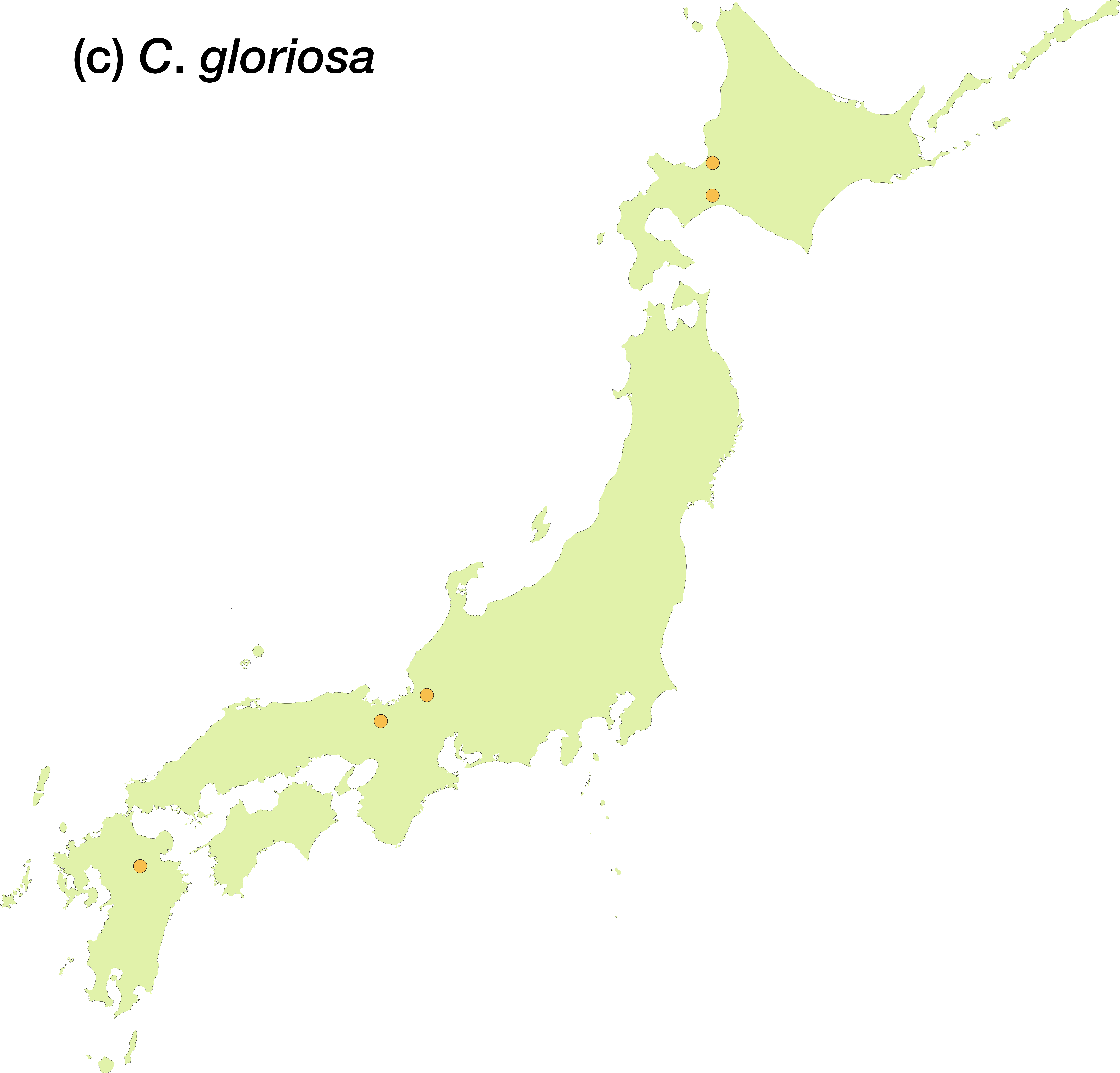
(c) *C. gloriosa*

(d) *C. hidakensis*

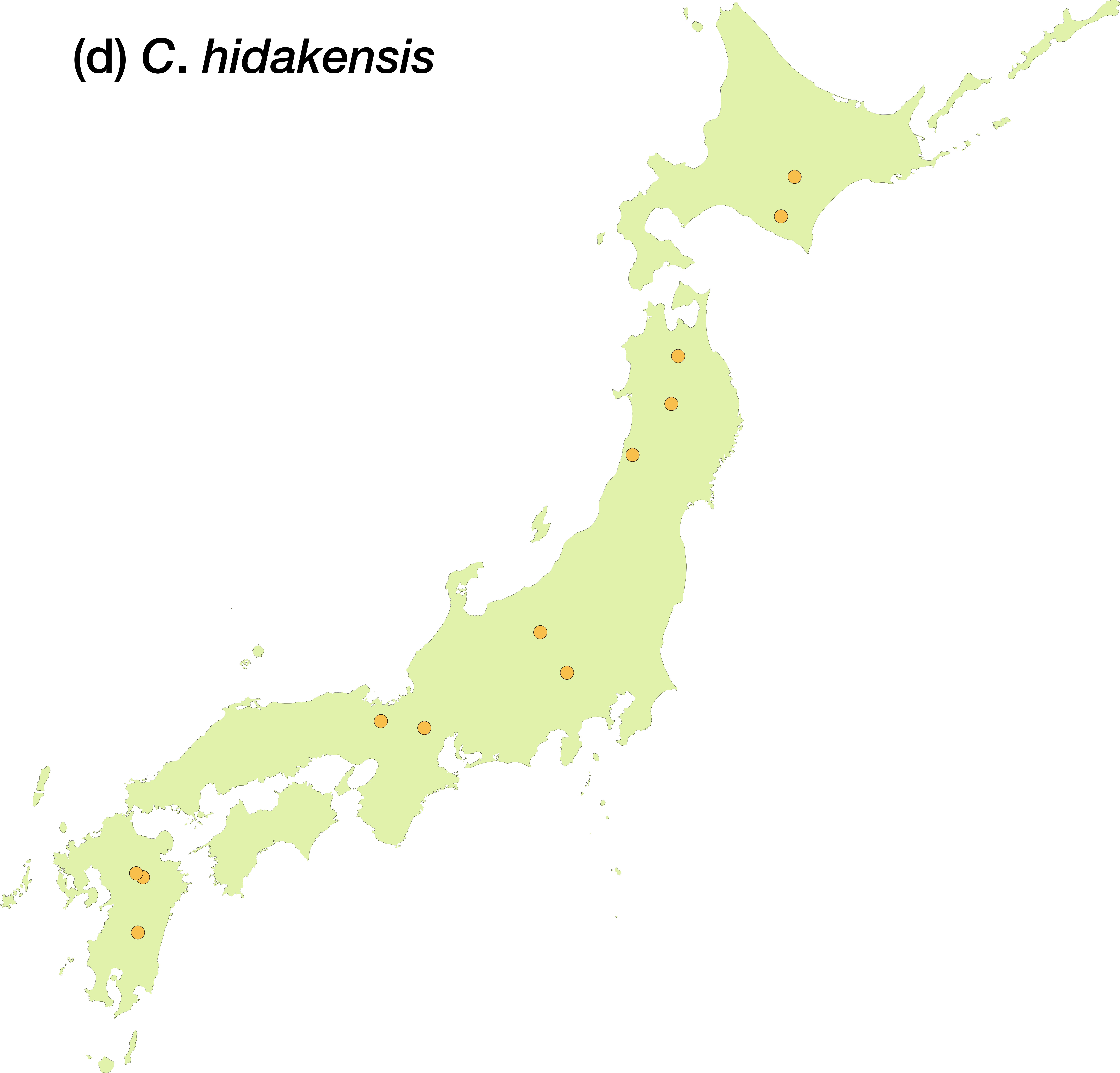

(e) *C. kisoensis*

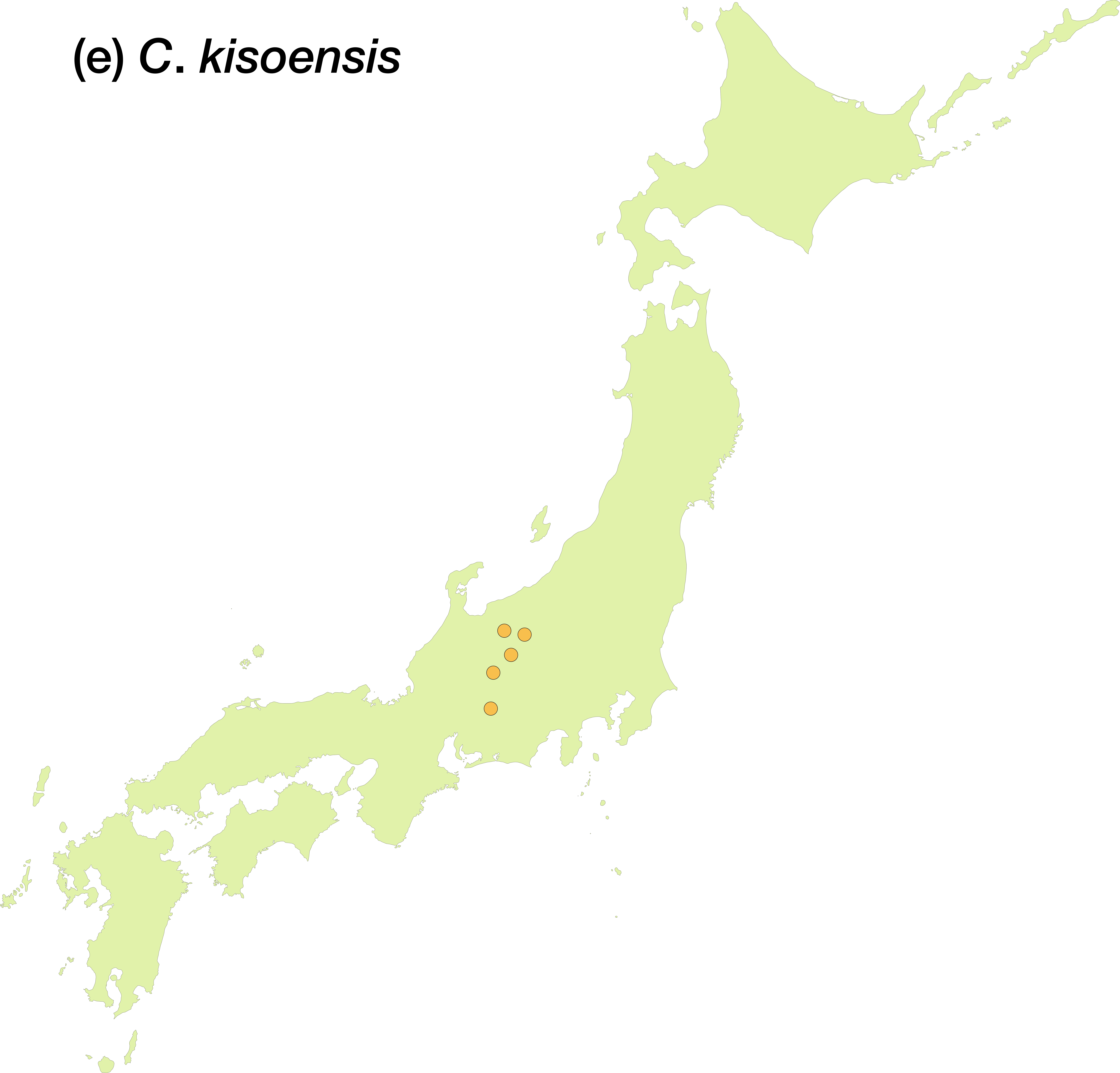

(f) *C. kurokoi*

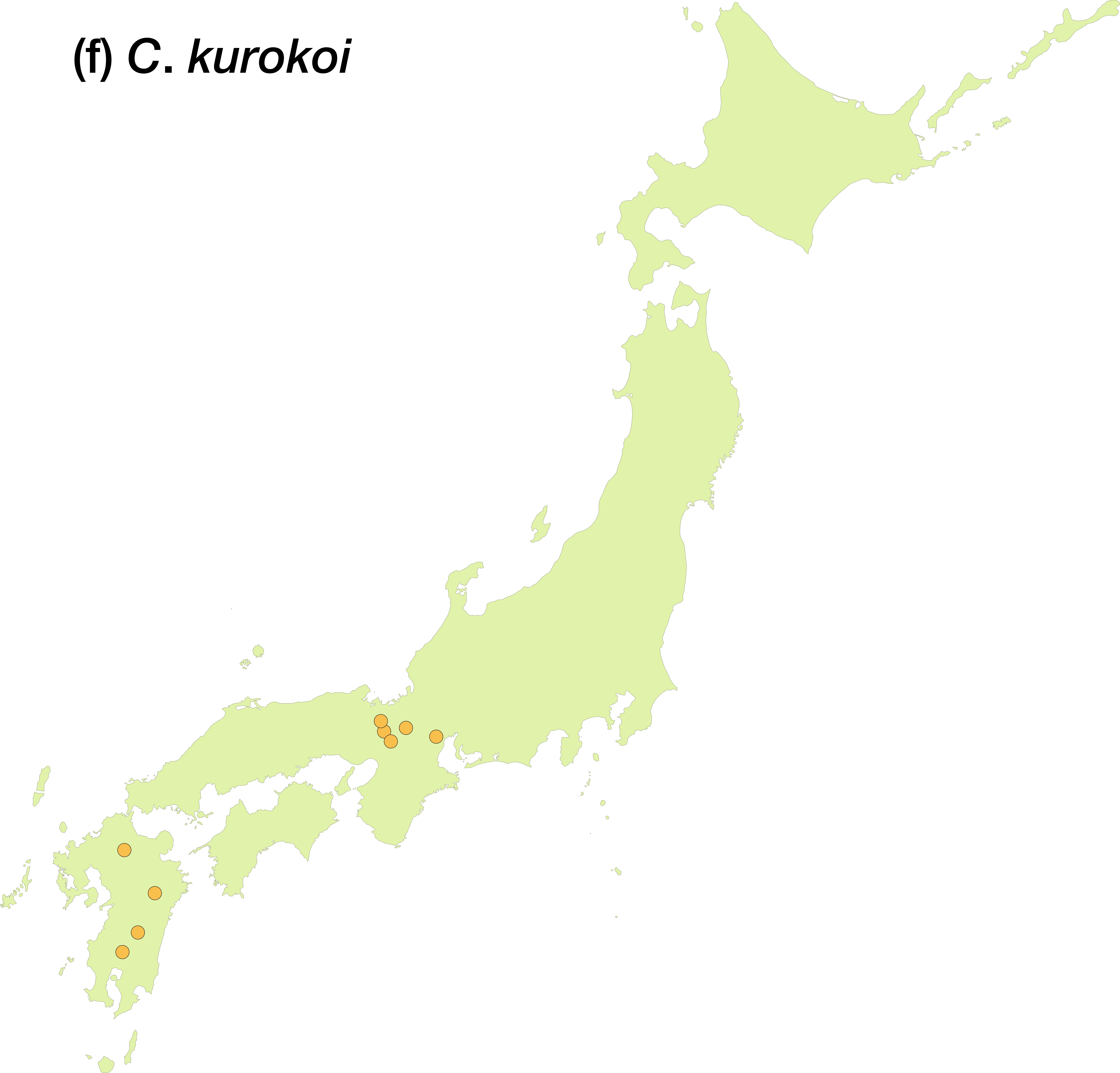

## (g) *C. monticola*

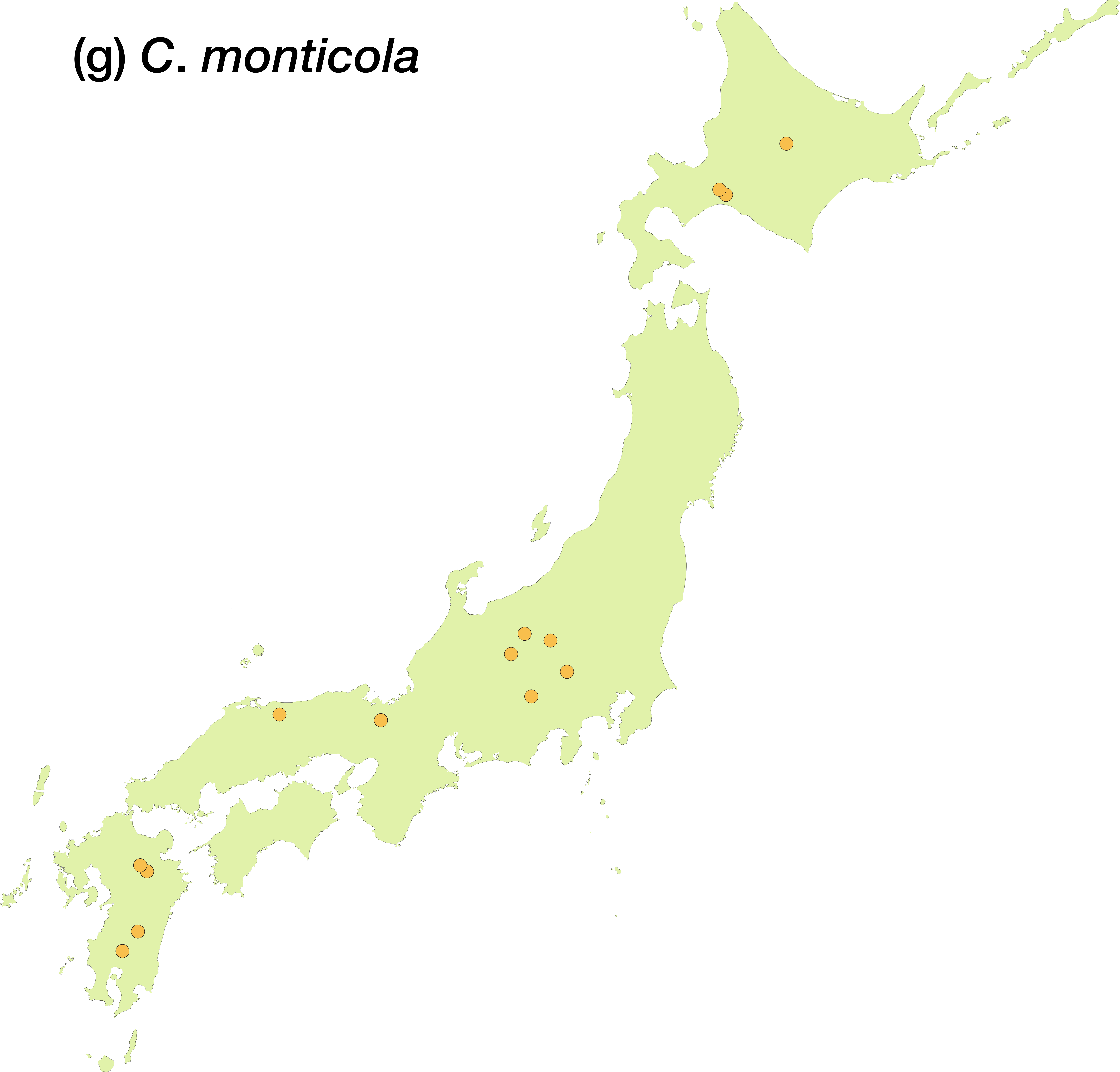

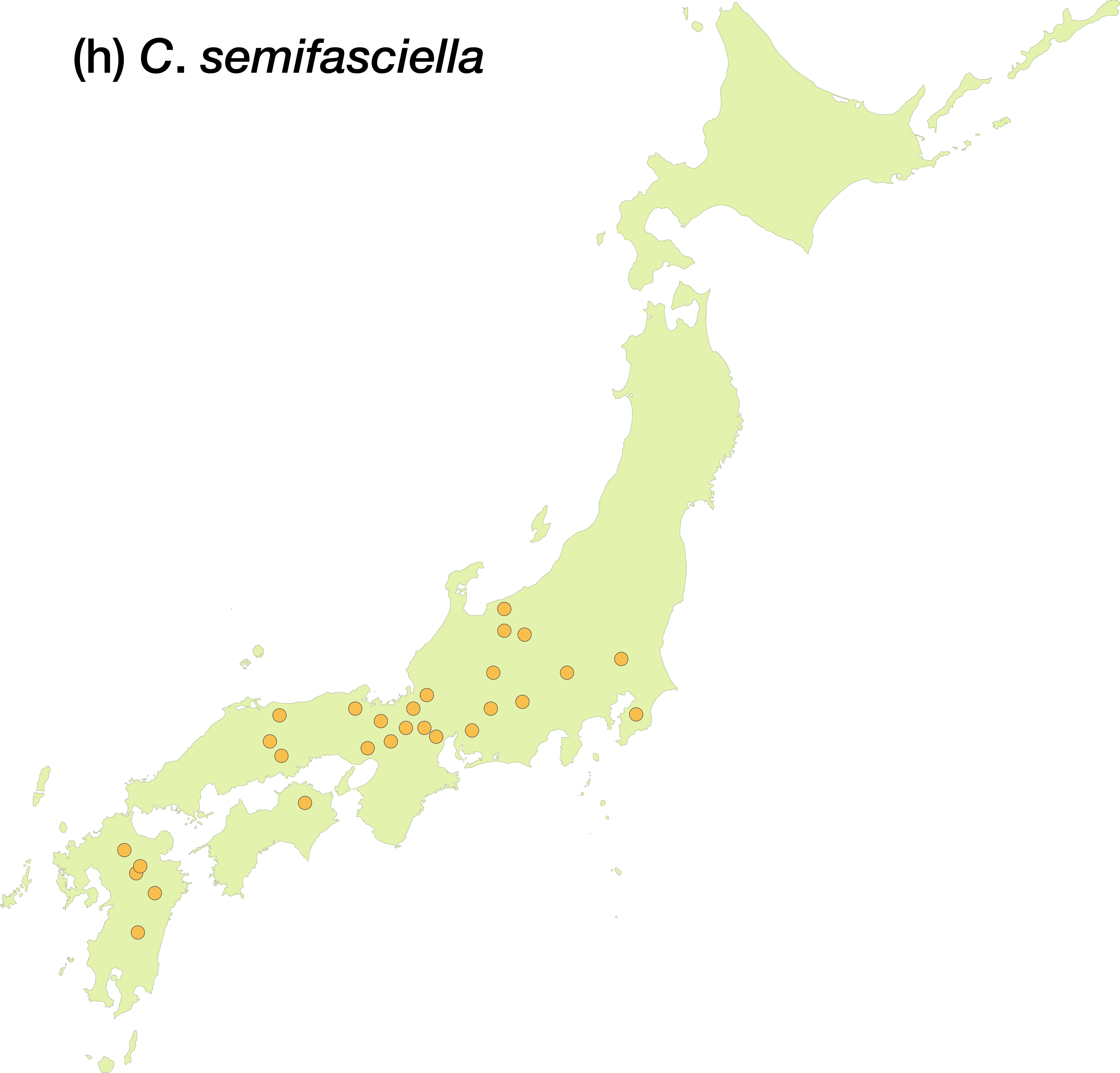
(h) *C. semifasciella*

## (i) *C. wakayamensis*

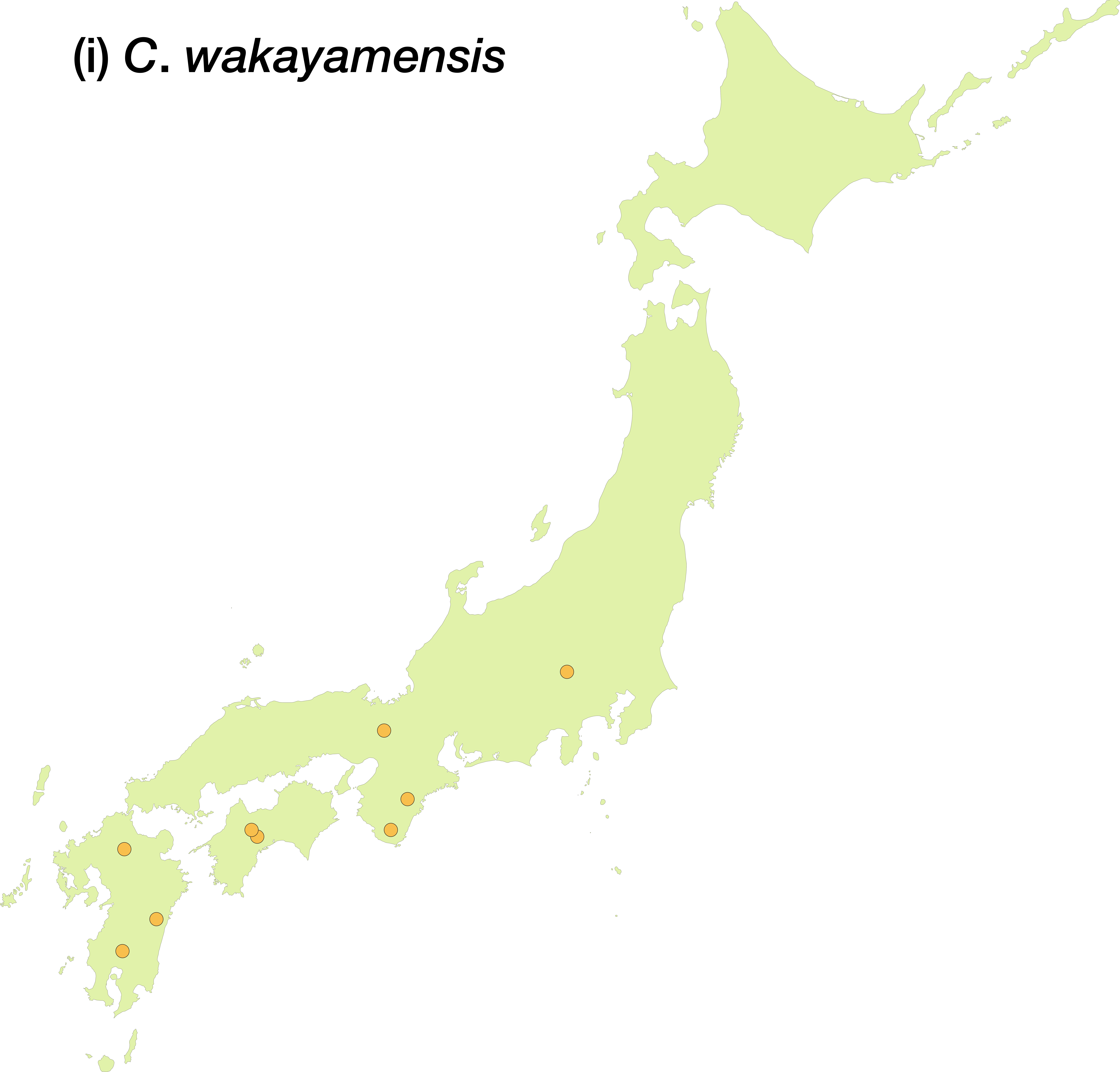

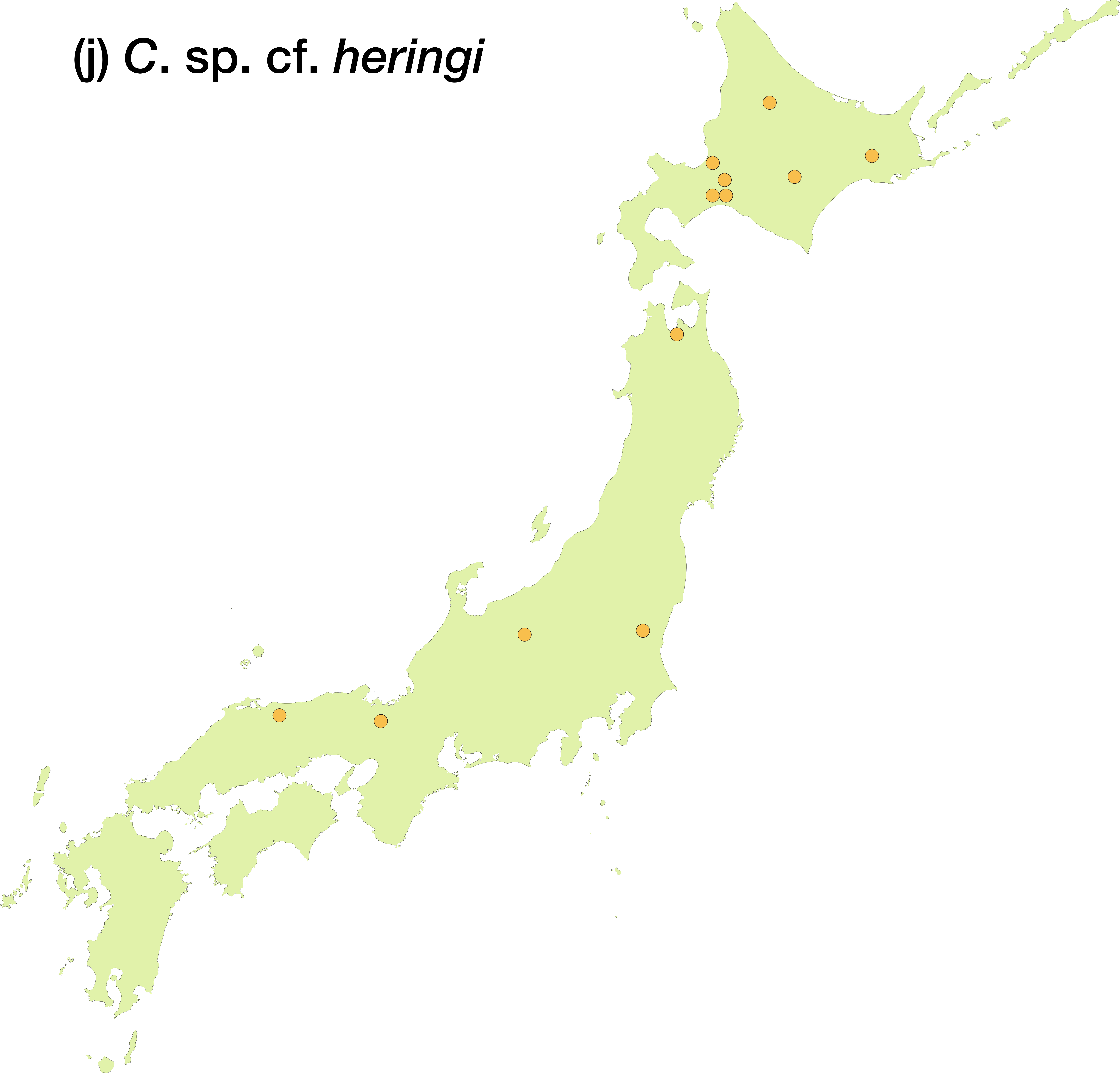
(j) *C.* sp. cf. *heringi*

(k) *C.* sp. cf. *yasudai*

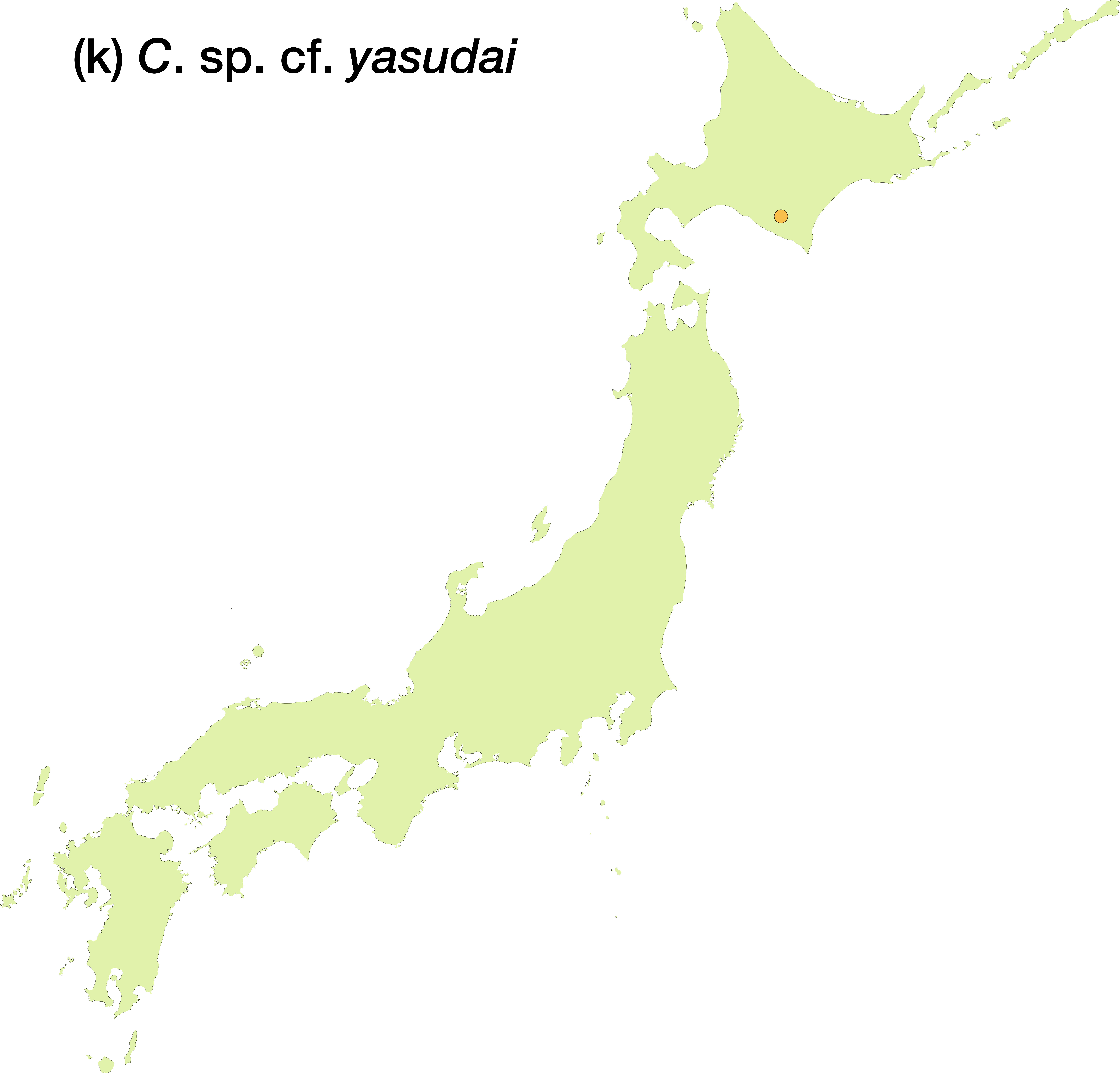

(l) *C.* sp. 1

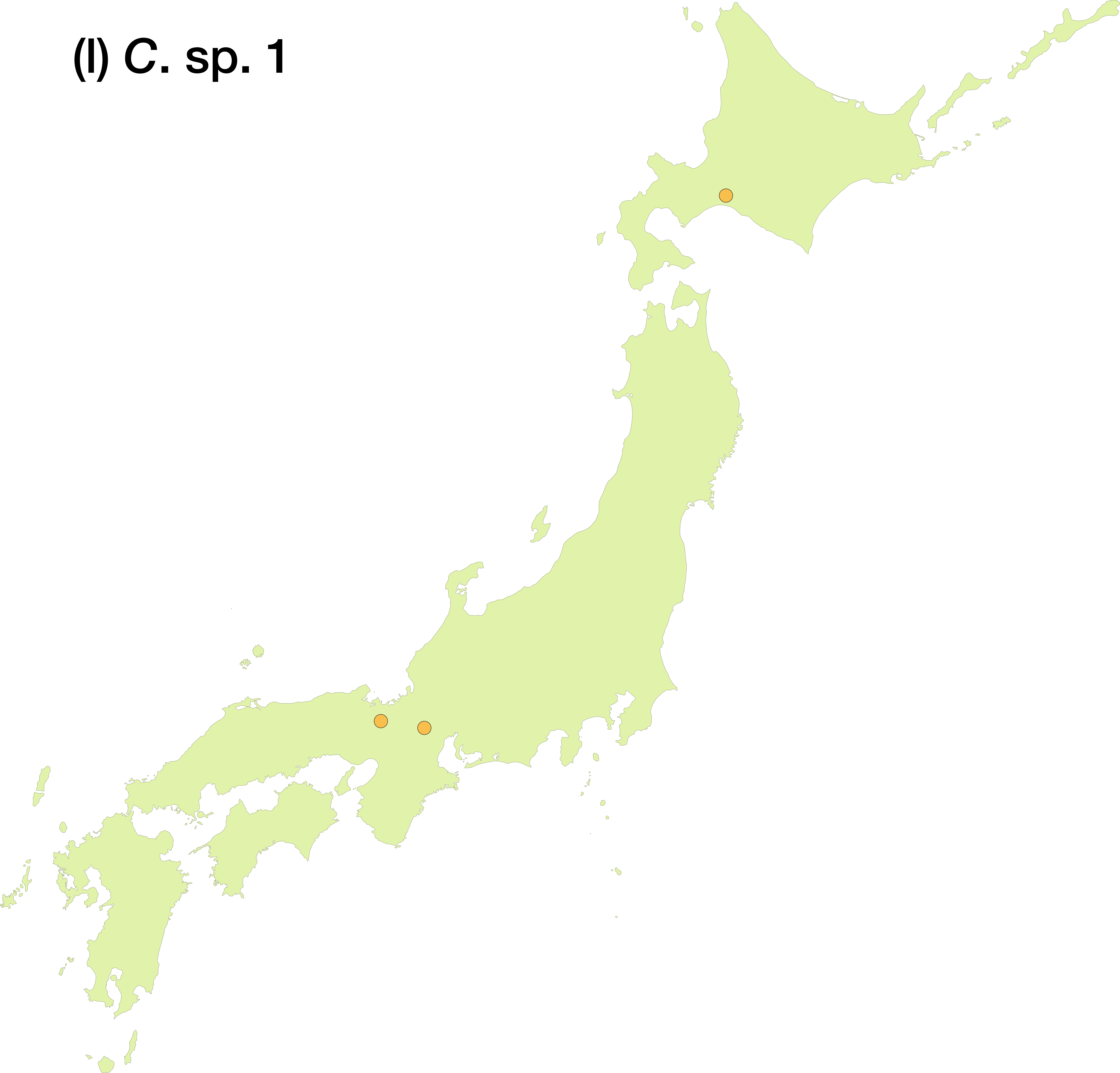

(m) *C.* sp. 2

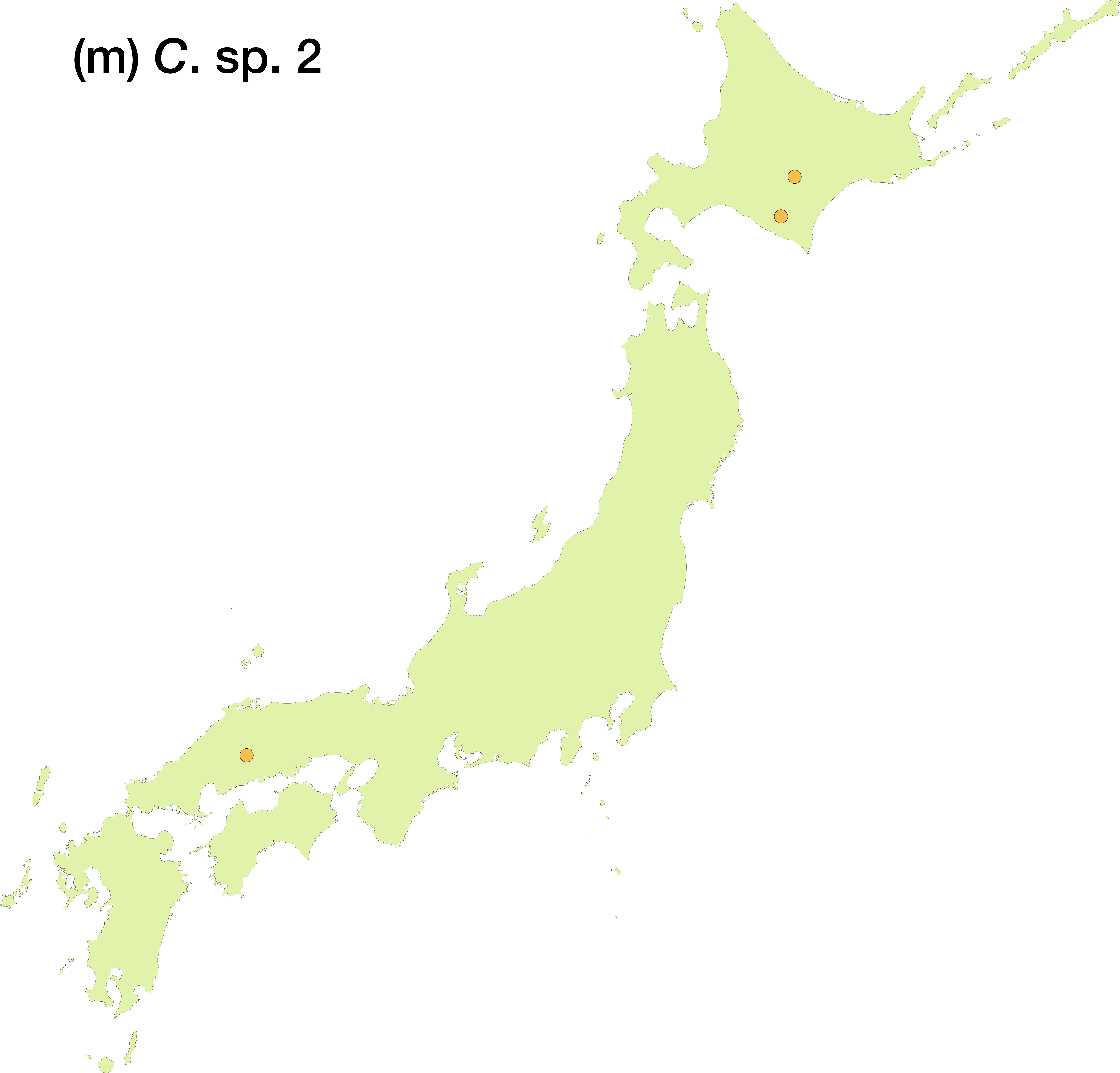

(n) *C.* sp. 3

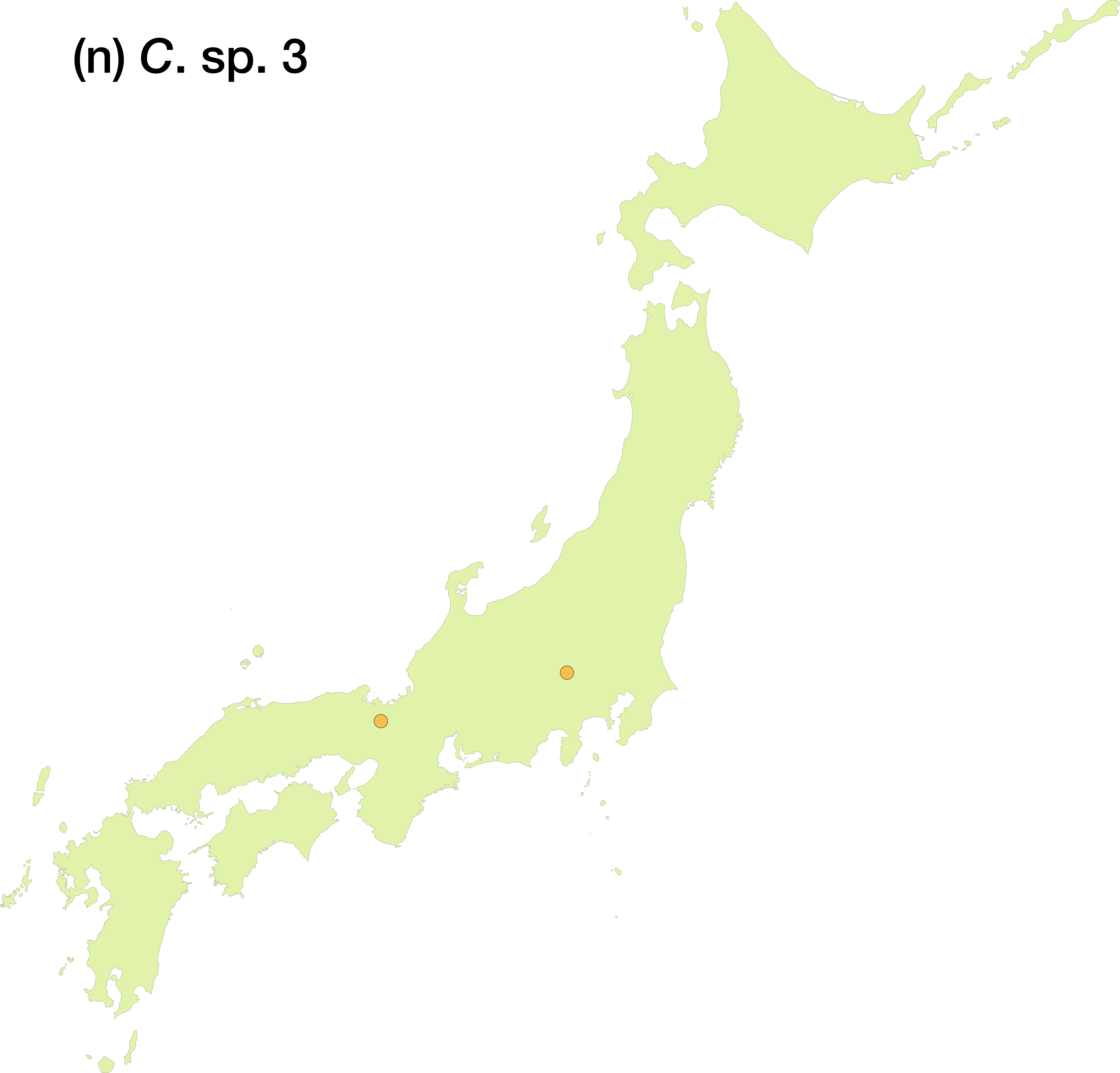

Supplement: Supplementary file 2 — Figure S2. Distributions of 14 Caloptilia moth species feeding on Acer. [file ECE3-6-4958-s002.pdf]
